# The clinical course of low back pain: a meta-analysis comparing outcomes in randomised clinical trials (RCTs) and observational studies

**DOI:** 10.1186/1471-2474-15-68

**Published:** 2014-03-07

**Authors:** Majid Artus, Danielle van der Windt, Kelvin P Jordan, Peter R Croft

**Affiliations:** 1 Arthritis Research UK Primary Care Centre, Primary Care Sciences, Keele University, Keele, Staffordshire ST5 5BG, UK

## Abstract

**Background:**

Evidence suggests that the course of low back pain (LBP) symptoms in randomised clinical trials (RCTs) follows a pattern of large improvement regardless of the type of treatment. A similar pattern was independently observed in observational studies. However, there is an assumption that the clinical course of symptoms is particularly influenced in RCTs by mere participation in the trials. To test this assumption, the aim of our study was to compare the course of LBP in RCTs and observational studies.

**Methods:**

Source of studies CENTRAL database for RCTs and MEDLINE, CINAHL, EMBASE and hand search of systematic reviews for cohort studies. Studies include individuals aged 18 or over, and concern non-specific LBP. Trials had to concern primary care treatments. Data were extracted on pain intensity. Meta-regression analysis was used to compare the pooled within-group change in pain in RCTs with that in cohort studies calculated as the standardised mean change (SMC).

**Results:**

70 RCTs and 19 cohort studies were included, out of 1134 and 653 identified respectively. LBP symptoms followed a similar course in RCTs and cohort studies: a rapid improvement in the first 6 weeks followed by a smaller further improvement until 52 weeks. There was no statistically significant difference in pooled SMC between RCTs and cohort studies at any time point:- 6 weeks: RCTs: SMC 1.0 (95% CI 0.9 to 1.0) and cohorts 1.2 (0.7to 1.7); 13 weeks: RCTs 1.2 (1.1 to 1.3) and cohorts 1.0 (0.8 to 1.3); 27 weeks: RCTs 1.1 (1.0 to 1.2) and cohorts 1.2 (0.8 to 1.7); 52 weeks: RCTs 0.9 (0.8 to 1.0) and cohorts 1.1 (0.8 to 1.6).

**Conclusions:**

The clinical course of LBP symptoms followed a pattern that was similar in RCTs and cohort observational studies. In addition to a shared ‘natural history’, enrolment of LBP patients in clinical studies is likely to provoke responses that reflect the nonspecific effects of seeking and receiving care, independent of the study design.

## Background

Well-conducted randomised clinical trials (RCTs) generally provide the strongest evidence for the effectiveness of treatments. RCTs on the effectiveness of treatments for non-specific low back pain have not found evidence for a clear superiority of any treatment [1-3]. Yet, low back pain symptoms tend to improve in RCTs regardless of the treatment provided. Such improvement seems to follow a pattern common to all treatment arms, of rapid early improvement within the first 6 weeks reaching a plateau over the following 12 months [4]. This is explained at least partly by the ‘natural history’ (i.e. the propensity for symptoms to improve without treatment). With the use of treatment this is referred to as the ‘clinical course’ of symptoms. The clinical course of back pain has been assessed in observational (cohort) studies [5,6]. It was also found to follow a pattern of general improvement that starts rapidly and plateaus over time. Although this suggests a similarity between RCTs and cohort studies, there is no clear evidence for this from direct comparison. More importantly, it is not clear whether the size of overall symptom improvement is the same in these two groups of studies. There is only a limited evidence for a direct comparison, mainly comparing RCTs with non-randomised trials and observational studies that included comparator groups [7].

There is an assumption that the course of symptoms in RCTs is different from that in cohort studies. It has been suggested that the mere participation in a trial influences the course of symptoms [8,9]. This might be explained by benefits perceived by participants and assumed to be related to the intensive assessment and monitoring. The so called ‘Hawthorne effect’ was quoted as an example of how individuals change behaviour due to the attention they receive from researchers. [10-12]. Although this is expected to apply to all studies, it might be relatively more pronounced in RCTs compared with cohort studies.

Another issue is whether participants in RCTs are in some way different from the average person presenting for care in usual clinical practice. Whether their willingness to be randomly allocated to a treatment or a placebo makes these individuals different from the average patient to whom the results of RCTs will be applied. If true, this raises the issue of whether participants in RCTs are less representative of the average patients compared with participants in observational studies in which patients are not randomised.

It is therefore important to establish the evidence for the similarity or otherwise, in the pattern and the size of back pain symptom improvement in these two types of studies. This would test the assumption that mere willingness to enrol in RCTs and be randomised to treatments would influence the clinical course of symptoms. This would have potentially important implications on interpreting the results of RCTs and their generalizability in clinical practice.

The aim of this systematic review and meta-analysis was to compare changes in low back pain symptoms over time in RCT participants with those of participants in observational cohort studies.

## Methods

### Criteria for inclusion

Included were studies (RCTs and prospective observational cohort studies) conducted for primary care treatment for LBP (e.g. analgesia, exercises, manipulation therapy) among individuals aged 18 or over. Studies had to provide baseline and follow-up data on the designated primary outcome measure of pain intensity, measured on a Numerical Rating Scale (NRS) or Visual Analogue Scale (VAS). Only studies published in English were included. Also excluded were studies conducted among patients with specific LBP (e.g. cancer or inflammatory arthritis), post-operative or post-traumatic back pain, or back pain associated with pregnancy or labour.

### Searching and selection of studies

To meet the specific aims of the study, the literature search did not have to be exhaustive, but to provide sufficiently large pool of studies. The Cochrane Central Register of Controlled Trials (CENTRAL) was therefore chosen as a sufficient data source for RCTs.. This search was an update (up to April 2012) of a strategy previously used and described elsewhere [4]. For observational studies, a literature search was conducted for the same time period using the databases of AMED, EMBASE, MEDLINE and CINAHL based on the keywords ‘low back pain’, ‘back pain’, ‘spinal pain’, ‘primary care’, ‘general practice’, ‘population’, ‘cohort’, ‘observational’, ‘prognosis’, predictor’ and ‘course’. The detailed search strategy is shown in Additional file 1. References accompanying relevant systematic reviews and included cohort studies were also hand-checked to identify additional eligible studies.

The literature search was conducted by MA and screening of citations/abstracts ad selection of RCTs and cohort studies applying the inclusion criteria was conducted by MA, DVdW & KPJ.

### Data extraction

The extracted data included:

1. Study characteristics (publication year, country of study, clinical setting, study design, sample size).

2. Participants’ characteristics (mean age;% female; duration of symptoms).

3. Interventions: name, dose and provider.

4. Outcome: baseline and follow up mean scores (and baseline standard deviation (SD)) for pain intensity.

### Analysis

Firstly, RCTs as a single group were compared with observational studies. Secondly, RCTs were sub-grouped into efficacy and pragmatic trials, based on whether the trial included a placebo, sham or no treatment, with such trials being grouped as efficacy trials. RCTs that included comparator treatment of usual care or waiting list arms were classified as pragmatic trials. To compare studies groups that are similar with regard to the type of treatment, a separate analysis was conducted to compare cohort studies with RCT arms that received ‘usual care’. Each RCT sub-group was compared separately with observational studies.

Pain intensity scores were converted to a zero to 100 scale (least to most severe) where necessary by multiplication. Meta-analysis using a random effects model was performed using STATA/IC 11 software to compute pooled mean pain intensity scores (and 95% confidence intervals) at baseline and follow up, separately for RCT treatment arms and for observational studies. Commonly used follow-up times of 6, 13, 27 and 52 weeks were selected for comparison. Data on other time points were considered to fall within the selected points if they were within a three-week range.

To compare the size of improvement in outcome scores in RCTs and observational studies, the standardized mean change (SMC) [13] was calculated for each RCT treatment arm and observational study by subtracting the follow-up mean outcome score from the baseline mean score and dividing by the standard deviation (SD) of baseline scores. Pooled SMCs were calculated using random effects meta-analysis. SMCs over 0.8 were considered large, 0.5 – 0.8 moderate and less than 0.5 small [14]. The 95% Confidence Intervals for SMCs were calculated using the formula described by Hozo et al. [15]. The variance (squared standard deviation, σ2) of response size was calculated using the following formula [15]:

$$\sigma 2=2\left(1‒\rho \right)/n\;\left[\left(n‒1\right)/\left(n‒3\right)\right]\;\left[1+n/2\left(1‒\rho \right)\delta \;2\right]‒\delta 2/\left[c\;\left(n‒1\right)\right]\;2$$

Where: c (n-1) approximates 1 - [3 / 4(n-1) –1], ρ is the population correlation between baseline and follow-up scores which was estimated as 0.5, n is sample size and δ is the SMC. Heterogeneity of studies’ estimates was assessed by computing *I*^*2*^ statistic [16], where zero indicates no variation between studies and 100% indicates that all variation is the result of variation between studies. Meta-regression analyses were conducted to test the significance of the difference in the size of SMCs between RCTs and observational studies at the selected follow up points.

## Results

### Included studies

**Figure 1:**
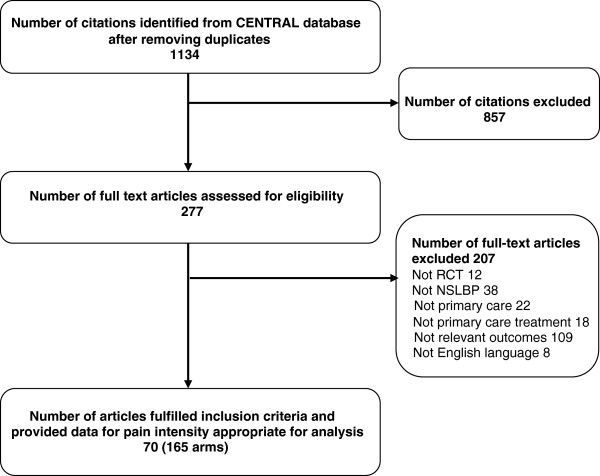
Identification and inclusion of RCTs in the systematic review.

**Figure 2:**
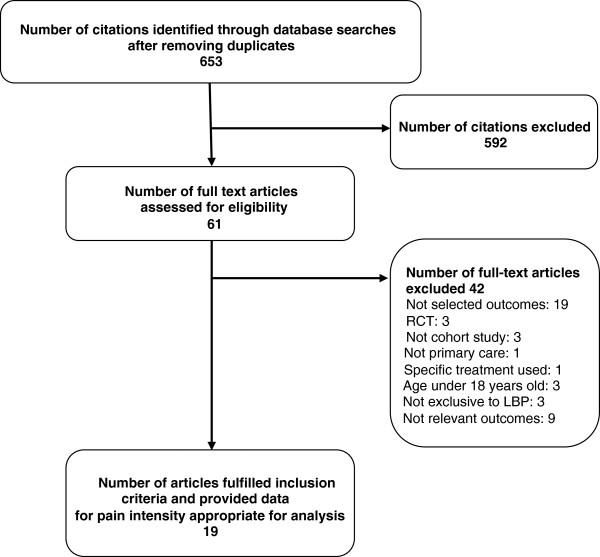
Identification and inclusion of observational cohort studies in the systematic review.

The updated search for RCTs yielded a total of 1134 citations of which papers for 70 RCTs (165 treatment arms) satisfied the inclusion criteria and provided pain intensity data useful for analysis (Figure 1). The search for observational studies yielded a total of 653 citations (Figure 2), and data for pain intensity useful for analysis were provided in 15 papers. Relevant data were obtained for further four papers by contacting authors, allowing analysis of pain intensity data from papers for a total of 19 observational studies.

### Characteristics of study setting and population

A list of the included RCTs and observational studies and their population characteristics are presented in Tables 1 &2. They were conducted in more than13 countries including the USA, Australia, and European countries during a period spanning two decades. They are comparable in terms of age distribution, gender composition and mean baseline pain intensity (Table 3). It appears that compared with observational studies, RCTs included a larger percentage of participants described as having chronic low back pain (57% in RCTs vs 11% in cohorts). However, these figures need to be interpreted with caution as observational studies often included a mixture of patients with acute and chronic back pain (19% in RCTs vs 63% in cohorts).

**Table 1:** Characteristics of included observational cohort studies (n 19)

**Author and country**	**Population and setting**	**Age, mean (y)**	**Female%**	**Type of back pain**	**Sample size**
Bakker et al., Netherlands [17]	GP consulters	41	48	Acute	97
Bekkering et al., Netherlands [18]	Physiotherapy consulters	45	52	Mixed	500
Carey et al., USA [19]	GP and chiropractic consulters	42	52	Acute	1628
Chenot et al., Germany [20]	GP consulters	44		Mixed	1342
Coste et al., France [21]	GP consulters	46	40	Acute	103
Demmelmeir et al., Sweden [22]	General population	42	55	Mixed	379
Dunn et al., UK [23]	GP consulters			Mixed	206
Grotle et al., Norway [24]	Primary care	38	55	Acute	123
Hass et al., Netherlands [25]	Community chiropractic clinics	43	53	Mixed	2780
Kovacs et al., Spain [26]	GP consulters	46	52	Mixed	648
McGuirk et al., Australia [27]	GP consulters	53	57	Acute	83
Miller et al., UK [28]	GP consulters	39	60	Mixed	211
Nyiendo et al., USA [29]	Medical and chiropractic clinics			Chronic	835
Perreault et al., Canada [30]	Physiotherapy departments	51		Mixed	78
Sefarlis et al., Sweden [31]	GP consulters	39		Acute	60
Sharma et al., USA [32]	Medical and chiropractic clinics consulters	40	50	Mixed	2872
Tamcan et al., Switzerland [33]	General population	42	50	Chronic	340
van Hoogan et al., Netherlands [34]	GP consulters	44	55	Mixed	443
van Tulder et al., Netherlands [35]	GP consulters	41	49	Mixed	368

**Table 2:** Characteristics of included RCTs (n 70)

**Author and country**	**Setting**	**Treatment**	**Age, mean (y)**	**Female (%)**	**Duration of back pain, mean (weeks)**	**Sample size of trial arms**
Albaladejo et al., Spain [36]	Primary care	Education & physiotherapy	51	68		100
		Education	51	63		139
		Usual GP care	53	72		109
Arribas et al., Spain [37]	National health centres	GDS physical therapy	39	64		78
		Electrotherapy	39	64		67
Bendix et al., Denmark [38]	General practice	Functional restoration (PT + OT + Psychological)	40	66		48
		Outpatient intensive physical training: Aerobics + strengthening exercises + fitness machines	43	69		51
Bronfort et al., USA [39]	College outpatient clinic	Spinal manipulation & trunk strengthening exercise	41	54	156	71
		NSAID & Trunk strengthening exercise	40	44	104	52
		Spinal manipulation & Stretching exercise	41	39	120	51
Bronfort et al., USA [40]	Physical therapy clinic	Supervised exercises	45	57	249	100
		Chiropractic	45	66	250	100
		Home exercises	46	58	250	101
Browder et al., USA [41]	Physical therapy clinics	Extension orientated exercises	40	31	9	26
		Strengthening exercises	38	32	9	22
Burton et al., UK [42]	General practice	The Back Book + usual care (GP & osteopathic care)		11		83
		The traditional Handy Hints & usual care (GP & osteopathic care)		12		79
Cambron et al., USA [43]	Chiropractic clinic + hospital clinic + General population	Chiropractic flexion distraction procedure	42	34		123
		Active trunk exercise program	41	41		112
Cecchi et al., Italy [44]	Rehabilitation department	Spinal manipulation	58	69		70
		Individual physiotherapy	61	61		70
		Back school	58	70		70
Chan et al., Hong Kong [45]	Physiotherapy	Aerobic training	47	79	54	24
		Usual physiotherapy	46	77	63	22
Chang et al., Taiwan [46]	General population	Piroxicam sachet	34	30		23
		Piroxicam tablets	34	26		19
Chok et al., Singapore [47]	Physiotherapy + Orthopaedic clinics + A/E	Physical therapy (endurance exercise at the PT department) + back hot pack	38	20	4	38
		Back hot pack (Home)	34	29	4	28
Costa et al., Australia [48]	Physical therapy clinics	Exercise	55	58	335	77
		Detuned diathermy and detuned USS	53	62	328	77
Constant et al., France [49]	General practice	Spa therapy & usual GP care				63
		Waiting list group & usual GP care				63
Critchley et al., UK [50]	Physiotherapy department	Individual physiotherapy	45	59	275	71
		Spinal stabilisation	44	71	346	72
		Pain management	44	62	348	69
Di Cesare et al., Italy [51]	Physical therapy clinics	Trigger point mesotherapy	53	55	22	29
		Acupuncture point mesotherapy	53	55	21	33
Djavid et al., Iran [52]	Occupational clinic	Low level laser (LLL)	40	56	118	20
		LLL + exercise	38	37	110	21
		Placebo LLL + exercise	36	17	106	20
Dufour et al., Denmark [53]	Rheumatology clinics	Group based multidisciplinary therapy	41	57	514	142
		Individual therapist assisted exercises	41	56	540	144
Dundar et al., Turkey [54]	Physical therapy clinics	Aquatic exercise	35	47		32
		Land based exercise	35	48		33
Fritz et al., USA [55]	Physical therapy clinics	Traction plus EOT	42	55		31
		Extension orientated therapy (EOT)	41	58		33
Frost et al., UK [56]	Physiotherapy	Routine physiotherapy & advice book	42	58		144
		Advice from physiotherapist & advice book	40	47		142
Geisser et al., USA [57]	University spinal programme	Manual therapy & Specific exercise (self corrections, stretching, strengthening)	39	67	284	26
		Sham Manual therapy & Specific exercise	39	56	370	25
		Manual Therapy & Non-specific exercise	37	80	370	24
		Sham Manual Therapy & non-specific exercise	46	61	284	25
George et al., USA [58]	Physical therapy	Standard care physical therapy	37	53	4	32
		Fear-avoidance based physical therapy	40	62	4	34
Glasov et al., Australia [59]	General population	Laser acupuncture	58	95		45
		Sham laser	49	62		45
Glomsrod et al., Norway [60]	Physicians clinics and General population	Active back school (Lectures and back exercises)	41	65		37
		Usual medical care	39	57		35
Goldby et al., UK [61]	General practice + hospital physicians	Spinal stabilisation & Attending the back school	43	68		84
		Manual therapy & Attending the back school	41	70		89
		Education (Booklet: Back in action) & Attending the back school	42	68		40
Hay et al., UK [62]	General practice	A brief programme of pain management (general fitness and exercise at clinic and home, explanation about pain mechanisms, distress, encouragement of positive coping strategies, overcoming fear of “hurt = harm”, and implementation of a graded return to usual activities)	40	50		201
		Physiotherapy including manual therapy techniques	41	55		201
Heymans et al., Netherlands [63]	Occupational healthcare	Usual Dutch occupational physician care	41	17	35	103
		Low intensity back school	41	22	35	98
		High intensity back school	40	23	35	98
Hseih et al., USA [64]	General population	Joint manipulation & myofascial therapy	48	33	12	52
		Joint manipulation	47	33	12	48
		Myofascial therapy	49	33	12	51
		Back school	48	40	11	48
Hurley et al., UK [65]	Physiotherapy + General practice + self referral	Manipulation therapy (Passively move intervertebral joint within or beyond its range)	40	57	8	80
		Interferential therapy (Electrical stimulation)	40	62	8	80
		Manipulation & interferential therapy	41	60	8	80
Hurwitz et al., USA [66]	Managed care facility	Chiropractic care only	52	49		169
		Chiropractic care & physical modalities (Heat/cold, USS)	54	58		172
		Medical care (excluding physical treatment) only	49	47		170
		Medical care & physical modalities (Heat/cold, USS)	49	54		170
Hurwitz et al., USA [67]	Network of healthcare	Chiropractic care only	52	49		340
		Chiropractic care & physical modalities (Heat/cold, USS)	53	58		340
Jellema et al., Netherlands [68]	General practice	Minimal intervention strategy (Assessing psychosocial risks, providing information on back pain and treatments & advice on self care)	43	48	2	143
		Usual GP care	42	47	2	171
Kaapa H., Finland [69]	Occupational healthcare	Multidisciplinary rehabilitation: guided, group programme. : CBT, relaxation, back school education & physical therapy	46	100	72	59
		Individual physiotherapy	47	100	63	61
Kankaanpaa, Finland [70]	Occupational healthcare	Active rehabilitation: guided exercises in a dept + behavioural support	40	34		30
		Passive treatment: which they considered as minor to the active arm, e.g. massage and thermal treatment	39	33		24
Kapitza et al., Germany [71]	General population	Contingent biofeedback	53	67	655	21
		Non-contingent biofeedback (placebo)	54	62	800	21
Karjalainen et al., 2003 & 2004, Finlands [72,73]	General practice	Mini-intervention (Specific back exercises, reduce patient concerns & encourage physical activity)	44	59		56
		Mini-intervention & worksite visit	44	57		51
		Usual GP care	43	60		57
Kennedy et al., UK [74]	Primary care	Acupuncture + back book	47	46		24
		Sham acupuncture + back book	45	58		24
Kerr et al., UK [75]	General practice	Acupuncture	43	50	86	30
		Placebo TENS (non-functioning)	43	65	73	30
Kovacs et al., Spain [76]	Nursing home consulters	Back book education	80	66		233
		Back guide education	81	63		199
		Pamphlet with cardiovascular health advice	80	64		241
Kuukkanen et al., Finland [77]	Occupational healthcare	Intensive training: intensive progressive exercises guided at the gym + home exercises		62		29
		Home exercise only: same as intensive, but unguided		48		29
		Control: usual activities, no trial exercises		54		28
Leclaire et al., Canada [78]	Private physiatrist clinic	Standard care (rest, analgesics, physio) & Swedish back school	32	43		82
		Standard care (rest, analgesics, physio)	32	41		86
Lindstrom et al., Sweden [79]	Occupational healthcare	Swedish back school & workplace visit + graded exercise (CBT approach)		24		51
		Usual care: rest& analgesics & physical treatment		38		52
Linton et al., Sweden [80]	General practice + general population	Back pain pamphlet	45	71		70
		Comprehensive information package	44	74		66
		CBT intervention	44	70		107
Luijsterburg et al., Netherlands [81]	Primary care	Physical therpay + GP care	42	57		67
		Usual GP care	43	40		68
Machado et al., Brazil [82]	Physiotherapy	Psychotherapy	45	81	356	16
		Exercise	42	59	206	17
Mannion et al., 1999 & 2001, Finland [83,84]	General population	Modern active individual physiotherapy: strengthening, coordination and aerobics exercises, instructions on ergonomic principles + home exercises	46	61	520	46
		Muscle reconditioning on training devices (small groups)	45	54	504	47
		Low impact aerobic/stretching (groups)	44	55	676	44
Maul et al., Switzerland [85]	Occupational healthcare	Back school & exercise	38			97
		Back school	39			86
Mehling et al., USA [86]	General practice	Breath therapy	50	70	51	16
		Physical therapy: soft tissue mobilisation, joint mobilisation and exercises	49	58	57	12
Moseley L, Australia [87]	Physiotherapy + General practice	Physiotherapy	43	64		29
		Usual GP care	38	54		28
Niemisto et al., 2003 & 2005, Finland [88,89]	General population	Manipulation, exercise & physician consultation	37	55	312	102
		Physician consultation only	37	53	312	102
Nordeman et al., Sweden [90]	General practice + physical therapy dept	Early access to physio (Individualised, exercise, advice, group education)	39	63		32
		Waiting list control	41	50		28
Paatelma et al., Finland [91]	Occupational clinic	Orthopaedic manual therapy	44	42		45
		McKenzie technique	44	29		52
		Advice only	44	35		37
Peloso et al., USA [92]	Outpatients	Tramadol & Acetamenophen combination tablets 375/325 2 PRN	58	64		167
		Placebo tablets 2 PRN	58	61		169
Rantonen et al., Finland [93]	Occupational clinic	Physical therapy	44	35	676	43
		Exercise	45	28	520	43
		Back book education	44	32	728	40
Rasmussen-Barr et al., Sweden [94]	Physiotherapy	Graded exercises	37	50	468	36
		Advice and walking	40	50	572	35
Rasmussen-Barr et al., Sweden [95]	Physiotherapy	Stabilizing training (Individual) (Cognitive + stabilisation of spinal muscles)	39	70		24
		Manual treatment (Individual) (Other muscles exercises, no manipulation)	37	78		23
Rittweger et al., Germany [96]	General population	Isodynamic lumbar extension	50	44	603	30
		Vibration exercise (On a machine with a vibrating platform)	54	52	754	30
Ritvanen et al., Finland [97]	General population	Traditional chiropractic bone setting	41	45		33
		Physical therapy	42	43		28
Rossignol et al., Canada [98]	Workers compensation board	Coordination of primary healthcare program	37	33		54
		Usual GP care	38	23		56
Sahin et al., Turkey [99]	Physical therapy clinics	Back school	47	75	30	75
		Physical therapy	51	78	32	75
Soukup et al., Norway [100]	General practice + general population + referrals	Mensediesk exercise group intervention	40	53	676	34
		Waiting list group	40	49	578	35
Staal et al., & Hlobil et al., Netherlands [101,102]	Occupational healthcare	Graded activity (Physiotherapy + OT)	39	5	9	67
		Usual OT care	37	8	8	67
Torstensen et al., Norway [103]	Social security offices	Medical exercise therapy (MET)	42	52		71
		Conventional physiotherapy (CP)	43	48		67
		Self exercise	40	51		70
Tsui et al., Hong Kong [104]	Physiotherapy	Electro-acupuncture & back exercise	40	76	39	14
		Electrical heat acupuncture + back exercise	39	71	54	14
		Back exercise only	41	62	50	14
Turner et al., USA [105]	General practice + physicians + general population	Relaxation training (group)				24
		Cognitive therapy (group)				23
		Cognitive therapy & Relaxation training (group)				25
		Waiting list control				30
Unsgaard-Tondel et al., Norway [106]	Primary care	Low load exercise	41	81	312	36
		High load sling exercise	43	64	468	36
		General exercise	36	65	312	37
van der Roer et al., Netherlands [107]	Physiotherapy	Intensive protocol training	42	55	54	60
		Guidelines based physiotherapy	42	48	47	54
Wand et al., UK [108]	General practice + A/E patients	Assess & Advice & Physiotherapy	34	44		43
		Assess & Advice & wait	35	55		51
Werners et al., Germany [109]	General practice	Interferential therapy: electrotherapy, to stimulate muscles fibres	38	43		68
		Motorised lumbar traction & massage	39	49		72
Yelland et al., Australia [110]	General practice	Glucose lignocaine injection	52	59	770	28
		Exercise (Alternating: flexion and extension of spine and hips)	49	55	718	26
		Saline injection	50	56	759	27
		Normal activity	51	58	733	29

**Table 3:** Comparison of population characteristics of included RCTs and observational cohort studies

		**Cohort studies**	**RCTs**^**a**^
**Publication year**		1994-2012	1993-2012
**Sample size, Median (range)**		368 (60, 2872)	128 (28, 681)^b^
			67 (12, 340)^c^
**Age, mean**^**d **^**(SD)**		43 (4.1)	44 (7.9)
**Female, mean percentage (SD)**		52 (4.8)	53 (16.9)
**Type of pain, n (%)**	**Acute**	5 (26)	34 (20)
	**Chronic**	2(11)	94 (57)
	**Mixed**	12(63)	31 (19)
	**Unclear**	0	6 (4)
**Baseline pain intensity, mean**^**d **^**(SD)**		49.6 (12.7)	49.9 (12.9)

^a^RCTs that provided data on pain intensity outcome. ^b^Sample size of RCT. ^c^Sample size of arm. ^d^Mean of all cohort/RCT means.

The setting of RCTs included general practice (18 RCTs), occupational health care departments (15 RCTs) and physiotherapy departments (19 RCTs). Eight trials were conducted among the general population and 10 in mixed settings. 13 RCTs (34 treatment arms) were classified by one of the authors (MA) as efficacy trials and the remaining 57 (131 treatment arms) as pragmatic trials. Eight RCTs included ‘usual care’ arms. The19 observational studies included consulters in general practice (11 studies) and other allied primary care services such as chiropractic clinics and physiotherapy departments, as well as cohorts sampled from the general population in two studies. All participants were described in the papers as receiving ‘usual’ or ‘standard care’.

### The course of pain intensity scores over time

**Figure 3:**
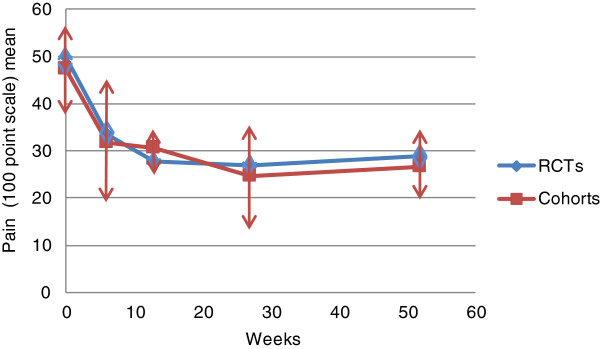
Pooled mean pain intensity scores (95% confidence interval) for the included RCTs and observational cohort studies from baseline to 52 week follow up.

Pooled mean pain intensity scores at baseline and follow up for RCTs and observational studies are presented in Figure 3 and Table 4. They show a similar pattern of symptom change over time in both groups. This is represented by a substantial rapid early improvement of mean pain intensity within the first 13 weeks of follow-up followed by a smaller further improvement over the follow-up period to 52 weeks.

**Table 4:** Pooled mean pain intensity scores (95% CI) for included RCTs and observational cohort studies using random effects meta-analysis

	**Baseline**	**6 weeks**	**13 weeks**	**27 weeks**	**52 weeks**
**RCTs**					
Pain	48.1 (45.8, 50.5)	34.1 (31.0, 37.2)	27.8 (25.1, 30.6)	26.4 (24.3, 28.6)	28.9 (25.7, 32.0)
Arms, n	165	58	94	97	78
Sample size*	10655	3577	6109	6640	4499
**Cohorts**					
Pain	47.3 (38.6, 56.0)	31.7 (18.5, 44.8)	30.7 (25.6, 35.8)	24.7 (12.9, 36.4)	26.7 (19.8, 33.6)
n	19	6	10	10	12
Sample size*	13096	6122	6848	5496	6284

*The total number of participants included in trials or cohort studies providing data for the analysis.

Regarding the size of symptom change over time, pooled SMCs (Table 5) confirm the substantial improvement in pain symptoms in both groups. These range from 0.9 to 1.2 for RCTs and from 1.0 to 1.2 for observational studies.

**Table 5:** Pooled estimates of SMCs (95% confidence interval) for pain intensity for included RCTs and observational cohort studies

	**Pooled SMCs (95% CI)**											
	**6 weeks**			**13 weeks**			**27 weeks**			**52 weeks**		
	**n**^**†**^		***I***^**2**^	**n**^**†**^		***I***^**2**^	**n**^**†**^		***I***^**2**^	**n**^**†**^		***I***^**2**^
**Cohorts**	6	1.2 (0.7, 1.7)	99	9	1.0 (0.8, 1.3)	99	9	1.2 (0.8, 1.7)	99	11	1.1 (0.8, 1.6)	99
**RCTs**	60	1.0 (0.9, 1.0)	99	94	1.2 (1.1, 1.3)	100	101	1.1 (1.0, 1.2)	100	78	0.9 (0.8, 1.0)	99
p-value*		0.651			0.735			0.878			0.721	
**Efficacy RCTs**	15	1.0 (0.9, 1.1)	99	13	1.2 (1.0, 1.4)	100	16	0.9 (0.7, 1.2)	100	14	0.7 (0.5, 0.8)	100
p-value**		0.663			0.549			0.574			0.104	
**Pragmatic RCTs**	43	1.0 (0.9, 1.1)	99	81	1.2 (1.1, 1.4)	100	81	1.2 (1.0,1.3)	100	64	0.9 (0.8, 1.1)	100
p-value***		0.628			0.466			0.899			0.642	
**Usual Care RCT arms**				8	1.2 (1.0, 1.3)	99	7	1.3 (1.7, 1.4)	99	7	1.0 (0.8, 1.2)	99

^†^Number of cohort studies and RCTs treatment arms. *Meta-regression comparison between cohort studies and RCTs. efficacy RCTs, **Meta-regression comparison between cohort studies and efficacy RCTs. ***Meta-regression comparison between cohort studies and pragmatic RCTs.

There was a large between-study variation in the sizes of pain improvement from baseline within both observational studies and RCT treatment arms demonstrated by the high *I*^2^ values (99%).

Meta-regression analysis showed no statistically significant difference in the change in pain intensity (SMC) between all RCTs and observational studies at any follow up point. There was also no statistically significant difference in the change in pain intensity when considering the two types of RCTs (pragmatic and efficacy) separately compared with observational studies. Comparing cohort studies and usual care arms of RCTs also did not show any difference in the pattern or course of LBP between these groups.

## Discussion

This study directly compared the course of non-specific low back pain symptoms in observational studies with RCTs on primary care treatments for back pain. The results showed no significant difference in the size of symptom improvement and the pattern of this improvement over time.

Investigating whether any difference is concentrated between observational studies and efficacy RCTs failed to show any difference in the size of symptom improvement. This was to test the assumption that compared with pragmatic RCTs, efficacy RCTs are characterised by higher level of attention and adherence to treatment protocol as well as stricter criteria for patient selection and inclusion [111,112]. Guidelines and tools are available to describe clinical trials as efficacy or pragmatic. The purpose of some of these tools is to inform trial design [111] while others are for the purpose of systematic reviews [112]. RCTs, however, are very rarely purely pragmatic or efficacy trials and could often be described along a continuum between these two ends and most include features of both with possible dominance of either. To satisfy the specific aims of our study related to the care and attention received in studies, the approach adopted was to describe trials that included placebo, sham or no treatment arms as efficacy trials.

A separate comparison between observational studies and the ‘usual treatment’ arms of RCTs was assumed to provide a comparison of groups receiving similar types of treatments. This comparison also failed to show any difference in the pattern or size of the clinical course of symptoms in these groups. This echoes what we have previously demonstrated of the absence of a significant difference in the pattern or size of symptom improvement in RCTs comparing usual care with active treatment arms [4].

One of the findings in this study was the large heterogeneity among cohort studies and RCT arms. Conducting meta-analysis in the presence of a large heterogeneity is potentially problematic. Using random effects model would have ameliorated this problem to an extent, but not completely. For this reason, the outcome of the meta-analysis will need to be interpreted within the specific context and aim of this study, namely to study the general trend of the clinical course of symptoms. The heterogeneity could be explained by a number of potential methodological as well as clinical characteristics. Formally studying such potential sources of heterogeneity is important and is beyond the aims of this study.

Meta-analyses comparing RCTs and observational studies have been conducted with varying aims including comparing treatment effects [111], adverse effects of treatments [112,113] and prognostic factors [114]. However, although the clinical course of low back pain has been studied in observational studies [10,11], we are not aware of a direct comparison with the clinical course of symptoms in RCTs. Furlan et al. [12] compared matching pairs of RCTs and non-randomised studies and included cohort studies but only those that had comparison groups. More significantly, the main aim of Furlan et al’s work was to compare RCTs with non-randomised studies regarding their methodological quality rather than to study the clinical course of symptoms.

A number of factors have been suggested to influence the course of symptoms in clinical trials, related to the participants (e.g. cultural background, health literacy) [115-117], the practitioner/researcher (e.g. communication skills and experience with the use of the treatment) [115,118] and the characteristics of the treatment (e.g. invasiveness, physical contact and psychological component) [119]. Another factor is suggested to relate to the actual enrolment in a trial. This is assumed to be related to the factual and perceived extensive care and attention provided in the trial - the ‘Hawthorne effect’, the ‘care effect’ or the unique strict adherence to the treatment protocol ‘protocol effect’. Such effects are assumed to contribute to extra improvement among participants in clinical trials compared with other studies or usual clinical practice [5].

The clinical course of back pain in observational studies might simply represent an extension of our earlier findings in RCTs [4]. This represents an average ‘general response to health care’ which dominates any individual responses to treatments. This general response overwhelms any additional effect of being in a trial, observational study or in fact seeking usual routine care. It is true that specific treatments are provided in RCTs as opposed to observational studies where no particular treatments are specified. In fact none of the observational studies included in our review included a specific treatment. However, conservative treatments for non-specific low back pain investigated in RCTs are not new but already available in clinical practice [1,3]. This might mean that expectations of novel and big effects among those participating in RCTs of back pain are not generally high.

Alternatively, differences may exist between RCTs and observational studies in the care and attention provided. But the effect on the clinical course of symptoms lies in outcomes other than those captured by pain intensity. Outcomes that may specifically represent components of a ‘trial effect’, and their measurement was beyond the scope of this paper.

Participants of observational studies are arguably similar to patients presenting in usual clinical practice. This means that our findings suggest that RCTs participants are not different from the average patients with regard to the clinical course of LBP. This challenges the assumption that participants in clinical trials are somehow different from the average patients. Or that their symptoms run a course that is to an extent influenced by mere participation in the trial. In other words, or findings would support the generalizability of the trials’ findings to patients in usual clinical practice. The findings also throws in doubt the assumption related to the effect of mere participation in a trial, although our study did not specifically aims to study this effect.

### Limitations

A large number of observational studies and RCTs on a wide range of treatments for non-specific low back pain were included to study the overall size of change in pain symptoms over time. The study, however, has a number of limitations.

For literature search, we adopted the same strategy that was adopted in a previous study conducted and published by the same group to examine the course of LBP in RCTs [4]. This was an updated access to the CENTRAL database. Although this might have limited the number of RCTs included in the study, it is unlikely that this represented a very large number that would have impacted the study outcome. Adopting the same strategy also provides the opportunity for a continuity of comparison between the two studies.

Also, as the aim of the study was to investigate the overall clinical course of LBP rather than to estimate the effectiveness of a particular treatment, an exhaustive inclusion of all trials on back pain treatments was not required. The aim was to have a large and representative pool of clinical trials that would vary sufficiently with respect to the types of treatments to achieve the objectives in this review and the CENTRAL database satisfied this aim. As a similar data base does not exist for observational cohort, a different search strategy was conducted for this group of studies.

The numbers of included RCTs and observational studies were not comparable. This might raise the concern that the outcome of the comparison is inaccurate. Although this is an arguably valid concern, the comparison with smaller subgroups of RCTs (efficacy RCTs and usual care arms) provided a more comparable numbers. The outcome of these comparisons confirmed the outcome of comparing the total groups of RCTs and cohort studies, which should help alleviate the related concerns.

The focus in our study was on pain intensity outcome using a Numerical Rating Scale (NRS) or Visual Analogue Scale (VAS). This was because of the lack of data on other outcome measures such as functional disability outcomes that would allow for a satisfactory comparison. The forced focus on one outcome measure in meta-analysis is common in systematic reviews of observational studies because of the lack of data on other outcome measures [11]. Excluding studies that did not provide data relevant to the analysis used in this study might have influenced our results. However, we have no evidence to suggest that this has led to systematic exclusion of studies with either large or small improvement in symptoms. We found in a previous review that the overall course of symptoms using functional disability outcomes (Roland Morris disability questionnaire, RMDQ and Oswestry Disability Inventory ODI) was similar to that when using pain intensity outcome [4].

## Conclusion

The course of back pain symptoms in observational studies follows a pattern that is similar to that in RCTs, notably in the size of the average improvement in pain intensity over time. This suggests that, in both types of studies, a general improvement in back pain symptoms and comparable responses to nonspecific effects related to seeking and receiving care occur regardless of the study design.

## Competing interest

The author(s) declare that they have no competing interests.

## Authors’ contribution

This study was part of a larger research project for the PhD conducted by MA, supervised by DvdW and KPJ. The PhD project was funded through an Arthritis Research UK Primary Care Fellowship, number 17890. All authors contributed equally to writing the article and all authors read and approved the final manuscript.

## Pre-publication history

The pre-publication history for this paper can be accessed here:

http://www.biomedcentral.com/1471-2474/15/68/prepub

## Supplementary Material

Additional file 1 Literature search strategy for observational cohort studies. Click here for file
