# Supplementary material for: The clinical course of low back pain: a meta-analysis comparing outcomes in randomised clinical trials (RCTs) and observational studies
Source: BMC Musculoskelet Disord. 2014 Mar 7;15:68. doi: 10.1186/1471-2474-15-68 (PMC4007531; doi:10.1186/1471-2474-15-68)
Supplement: Additional file 1 — Literature search strategy for observational cohort studies. [file 1471-2474-15-68-S1.docx]

**Additional file 1** Literature search strategy for observational cohort studies

**Back pain_Prognosis_Cohorts_Primary care (12/04/2012)**

1. exp Low back pain/ OR exp back pain/

2. exp Pain/

3. (back AND pain).ti,ab [Limit to: Core clinical journals (AIM) and Humans and English Language]

4. 1 AND 2 AND 3

5. exp Prognosis/

6. exp disease progression/

7. predict.ti,ab

8. factor.ti,ab

9. model.ti,ab

10. evolution.ti,ab

11. history.ti,ab

12. course.ti,ab

13. determinant.ti,ab

14. pattern.ti,ab

15. screen.ti,ab

16. long-term.ti.ab

17. progress.ti,ab

18. modif.ti,ab

19. mediate.ti,ab

20. OR/ 4-16

21. 4 AND 20

22. (epidemiology AND studies).ti,ab

23. exp Epidemiology studies/

24. cohort.ti,ab

25. retrospective.ti,ab

26. prospective.ti,ab

27. longitudinal.ti,ab

28. inception.ti,ab

29. observation.ti,ab

30. outcome.ti,ab

31. OR/ 22-30

32. 21 AND 31

33. exp Primary Health Care/

34. exp Family Practice/

35. exp Physicians, Family/

36. exp Community Health Services/

37. "General Practice".ti,ab

38. "family practice".ti,ab

39. "family physician".ti,ab

40. AMBULATORY CARE/

41. “ambulatory adj2 care”.ti,ab

42. OR/ 33-41

43. 32 AND 42
